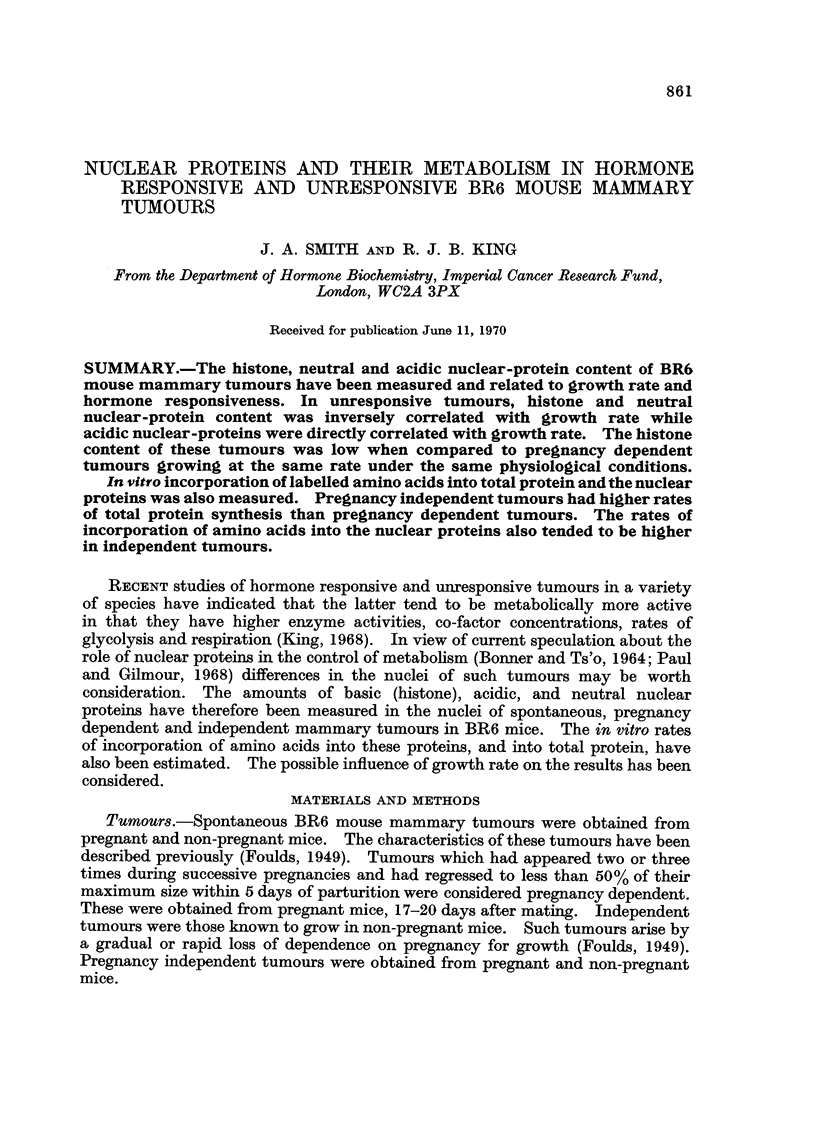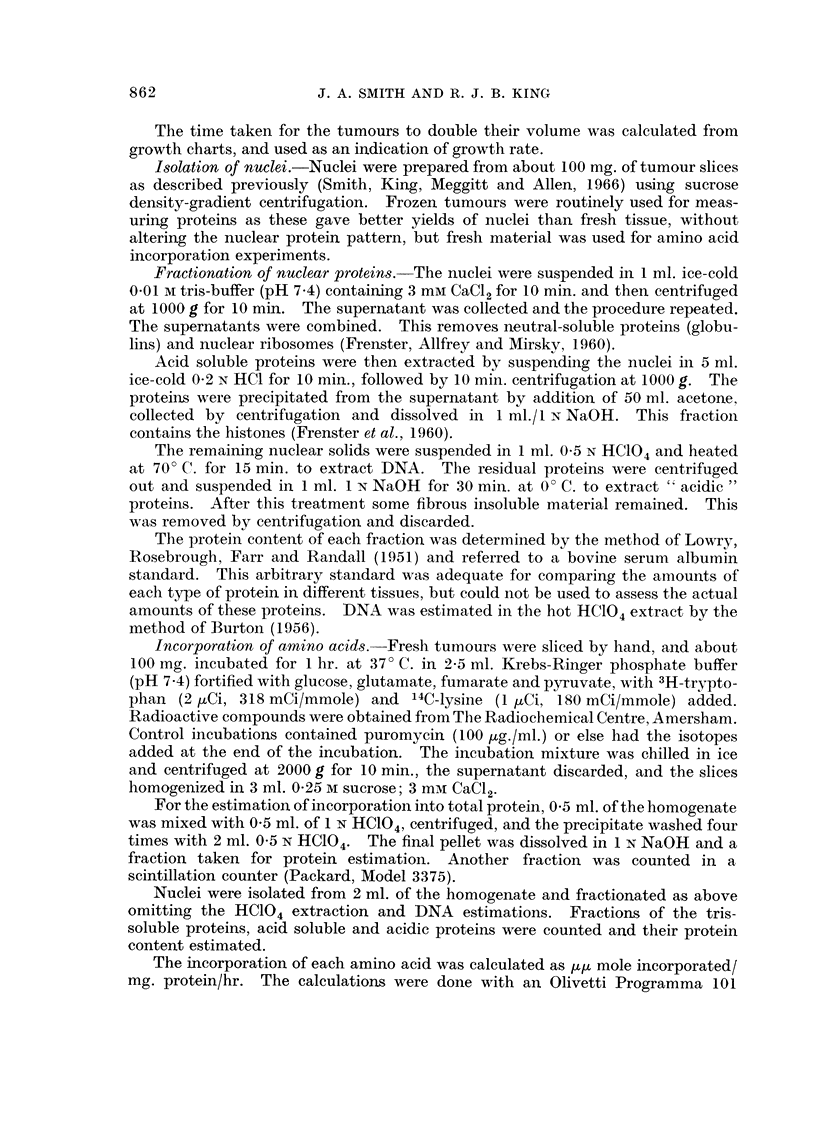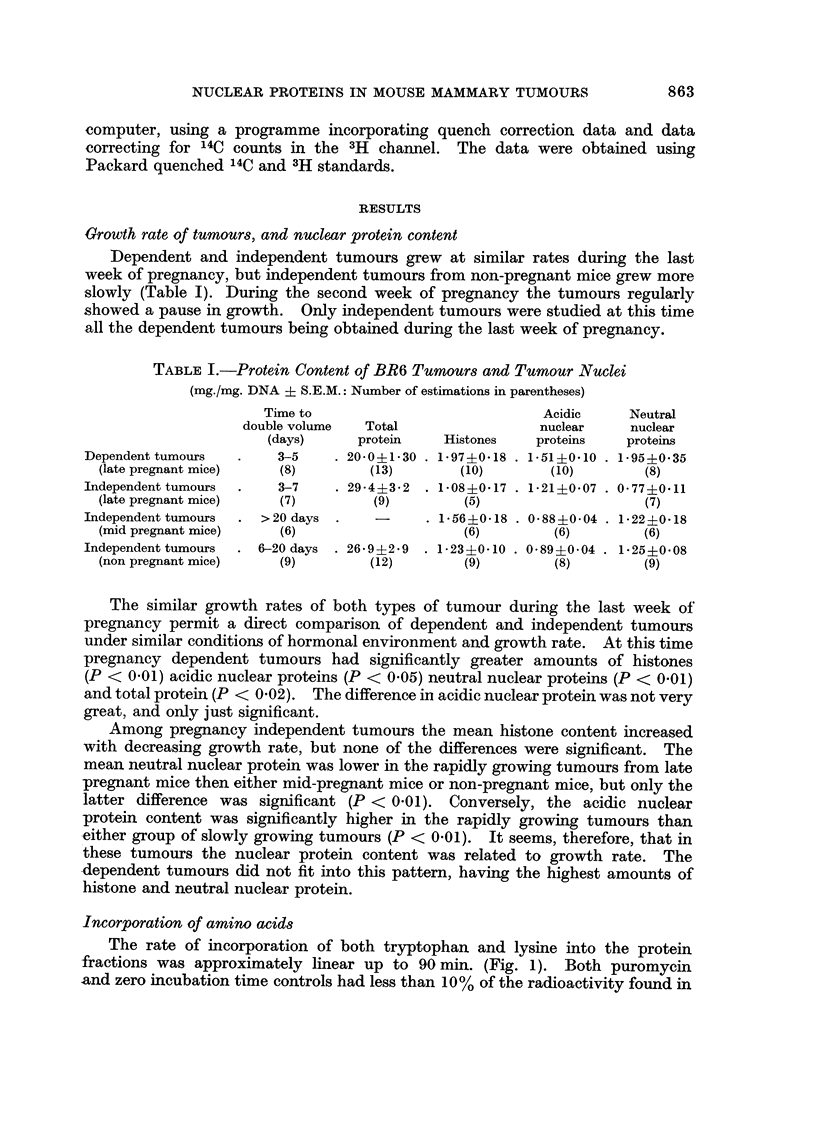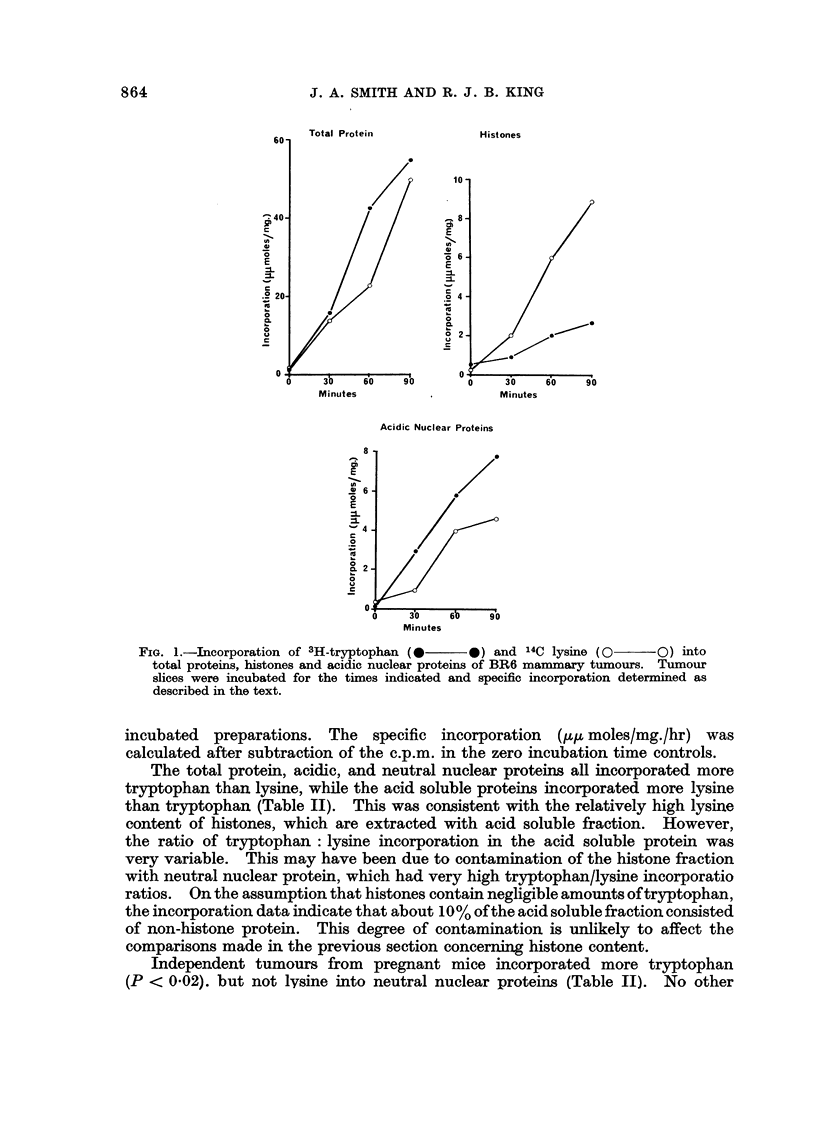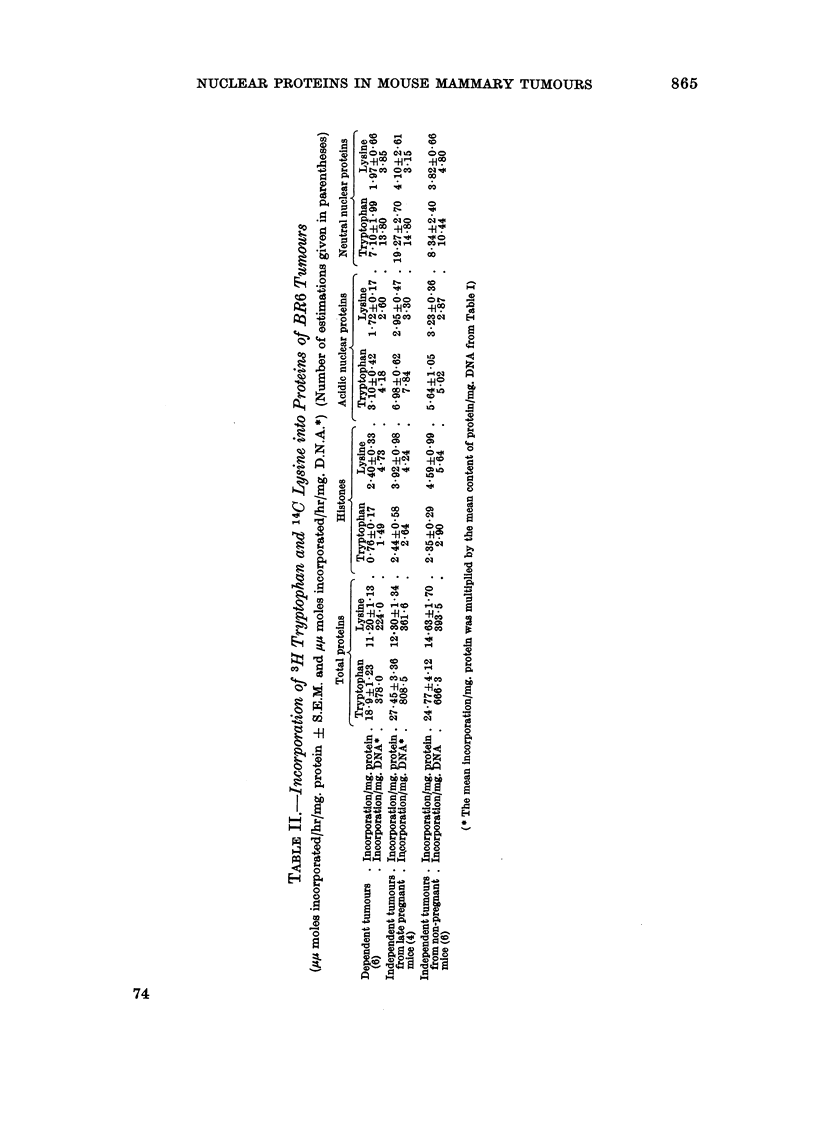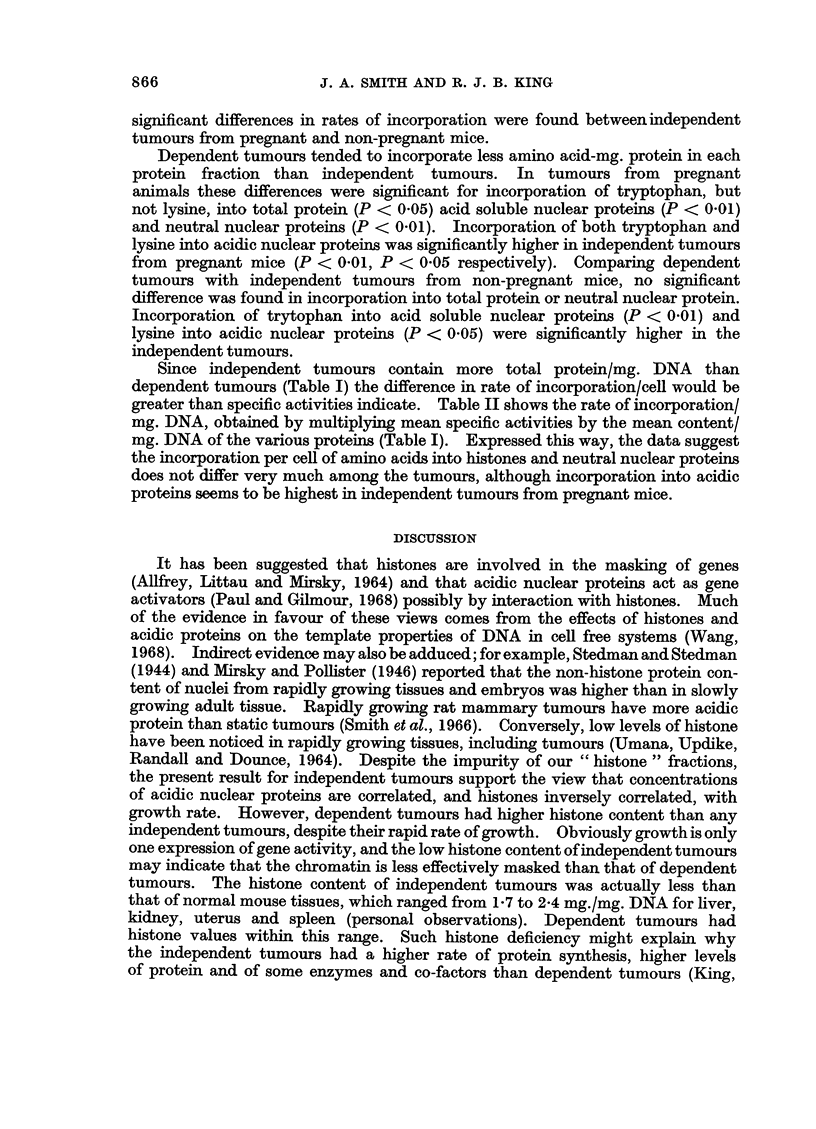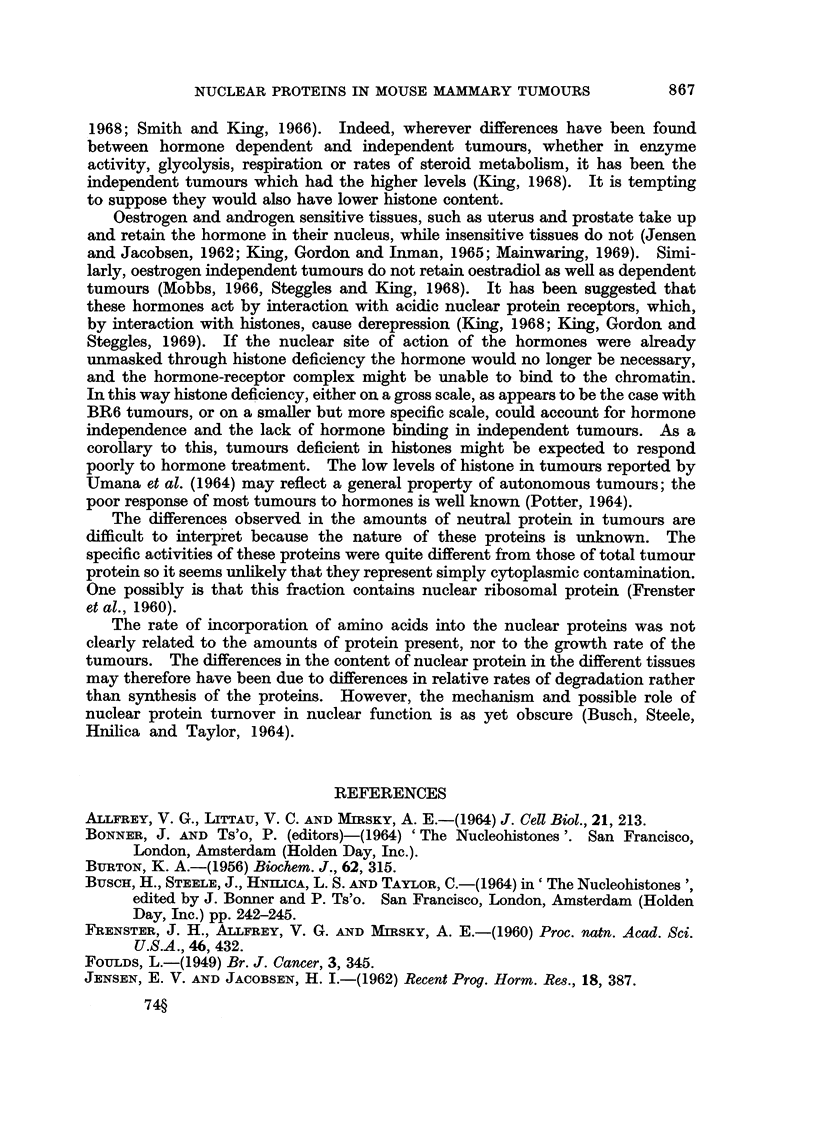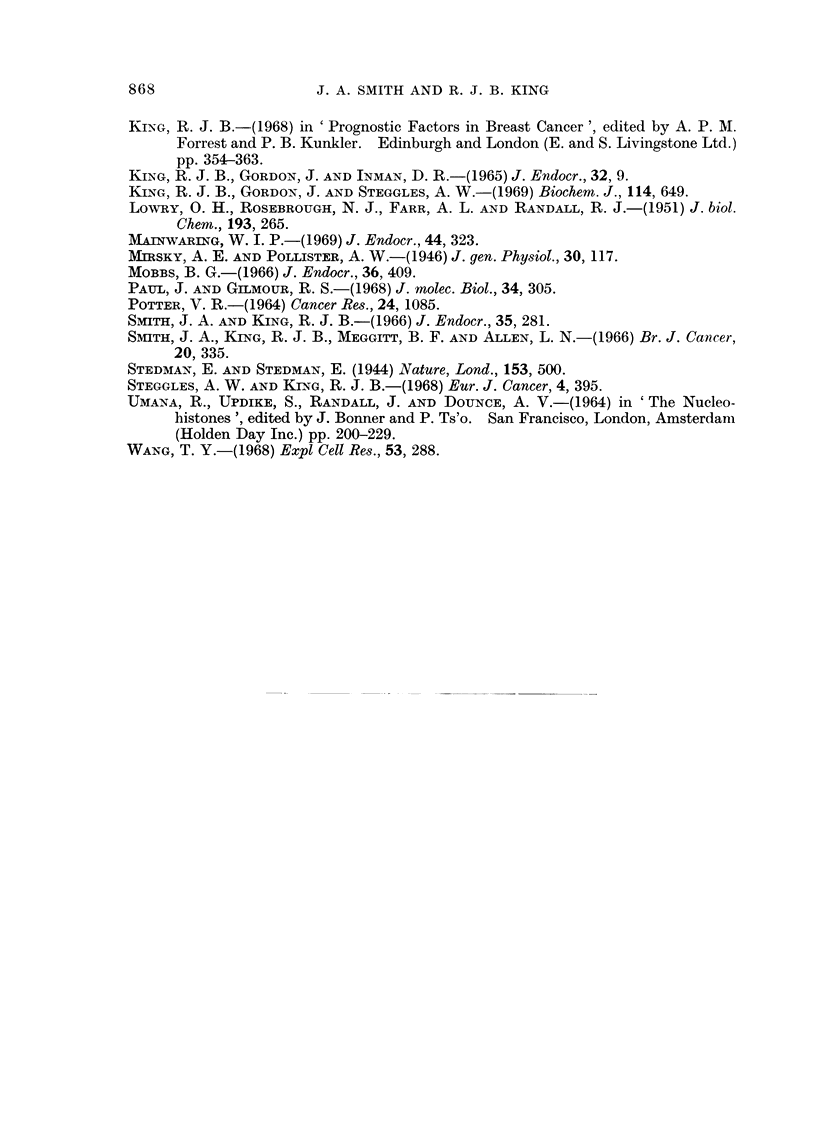# Nuclear Proteins and their Metabolism in Hormone Responsive and Unresponsive BR6 Mouse Mammary Tumours

**DOI:** 10.1038/bjc.1970.102

**Published:** 1970-12

**Authors:** J. A. Smith, R. J. B. King

## Abstract

The histone, neutral and acidic nuclear-protein content of BR6 mouse mammary tumours have been measured and related to growth rate and hormone responsiveness. In unresponsive tumours, histone and neutral nuclear-protein content was inversely correlated with growth rate while acidic nuclear-proteins were directly correlated with growth rate. The histone content of these tumours was low when compared to pregnancy dependent tumours growing at the same rate under the same physiological conditions.

*In vitro* incorporation of labelled amino acids into total protein and the nuclear proteins was also measured. Pregnancy independent tumours had higher rates of total protein synthesis than pregnancy dependent tumours The rates of incorporation of amino acids into the nuclear proteins also tended to be higher in independent tumours.


### NUCLEAR PROTEINS AND THEIR METABOLISM IN HORMONE RESPONSIVE AND UNRESPONSIVE BR6 MOUSE MAMMARY TUMOURS

J. A. SMITH AND R. J. B. KING

*From the Department of Hormone Biochemistry, Imperial Cancer Research Fund, London, WC2A 3PX*

Received for publication June 11, 1970

**SUMMARY.—The histone, neutral and acidic nuclear-protein content of BR6 mouse mammary tumours have been measured and related to growth rate and hormone responsiveness. In unresponsive tumours, histone and neutral nuclear-protein content was inversely correlated with growth rate while acidic nuclear-proteins were directly correlated with growth rate. The histone content of these tumours was low when compared to pregnancy dependent tumours growing at the same rate under the same physiological conditions.**

***In vitro* incorporation of labelled amino acids into total protein and the nuclear proteins was also measured. Pregnancy independent tumours had higher rates of total protein synthesis than pregnancy dependent tumours. The rates of incorporation of amino acids into the nuclear proteins also tended to be higher in independent tumours.**

RECENT studies of hormone responsive and unresponsive tumours in a variety of species have indicated that the latter tend to be metabolically more active in that they have higher enzyme activities, co-factor concentrations, rates of glycolysis and respiration (King, 1968). In view of current speculation about the role of nuclear proteins in the control of metabolism (Bonner and Ts'o, 1964; Paul and Gilmour, 1968) differences in the nuclei of such tumours may be worth consideration. The amounts of basic (histone), acidic, and neutral nuclear proteins have therefore been measured in the nuclei of spontaneous, pregnancy dependent and independent mammary tumours in BR6 mice. The *in vitro* rates of incorporation of amino acids into these proteins, and into total protein, have also been estimated. The possible influence of growth rate on the results has been considered.

### MATERIALS AND METHODS

*Tumours.*—Spontaneous BR6 mouse mammary tumours were obtained from pregnant and non-pregnant mice. The characteristics of these tumours have been described previously (Foulds, 1949). Tumours which had appeared two or three times during successive pregnancies and had regressed to less than 50% of their maximum size within 5 days of parturition were considered pregnancy dependent. These were obtained from pregnant mice, 17–20 days after mating. Independent tumours were those known to grow in non-pregnant mice. Such tumours arise by a gradual or rapid loss of dependence on pregnancy for growth (Foulds, 1949). Pregnancy independent tumours were obtained from pregnant and non-pregnant mice.

The time taken for the tumours to double their volume was calculated from growth charts, and used as an indication of growth rate.

*Isolation of nuclei.*—Nuclei were prepared from about 100 mg. of tumour slices as described previously (Smith, King, Meggitt and Allen, 1966) using sucrose density-gradient centrifugation. Frozen tumours were routinely used for measuring proteins as these gave better yields of nuclei than fresh tissue, without altering the nuclear protein pattern, but fresh material was used for amino acid incorporation experiments.

*Fractionation of nuclear proteins.*—The nuclei were suspended in 1 ml. ice-cold 0·01 M tris-buffer (pH 7·4) containing 3 mM $CaCl_2$ for 10 min. and then centrifuged at 1000 *g* for 10 min. The supernatant was collected and the procedure repeated. The supernatants were combined. This removes neutral-soluble proteins (globulins) and nuclear ribosomes (Frenster, Allfrey and Mirsky, 1960).

Acid soluble proteins were then extracted by suspending the nuclei in 5 ml. ice-cold 0·2 N HCl for 10 min., followed by 10 min. centrifugation at 1000 *g*. The proteins were precipitated from the supernatant by addition of 50 ml. acetone, collected by centrifugation and dissolved in 1 ml./1 N NaOH. This fraction contains the histones (Frenster *et al.*, 1960).

The remaining nuclear solids were suspended in 1 ml. 0·5 N $HClO_4$ and heated at 70° C. for 15 min. to extract DNA. The residual proteins were centrifuged out and suspended in 1 ml. 1 N NaOH for 30 min. at 0° C. to extract "acidic" proteins. After this treatment some fibrous insoluble material remained. This was removed by centrifugation and discarded.

The protein content of each fraction was determined by the method of Lowry, Rosebrough, Farr and Randall (1951) and referred to a bovine serum albumin standard. This arbitrary standard was adequate for comparing the amounts of each type of protein in different tissues, but could not be used to assess the actual amounts of these proteins. DNA was estimated in the hot $HClO_4$ extract by the method of Burton (1956).

*Incorporation of amino acids.*—Fresh tumours were sliced by hand, and about 100 mg. incubated for 1 hr. at 37° C. in 2·5 ml. Krebs-Ringer phosphate buffer (pH 7·4) fortified with glucose, glutamate, fumarate and pyruvate, with $^{3}H$-tryptophan (2 $\mu$Ci, 318 mCi/mmole) and $^{14}C$-lysine (1 $\mu$Ci, 180 mCi/mmole) added. Radioactive compounds were obtained from The Radiochemical Centre, Amersham. Control incubations contained puromycin (100 $\mu$g./ml.) or else had the isotopes added at the end of the incubation. The incubation mixture was chilled in ice and centrifuged at 2000 *g* for 10 min., the supernatant discarded, and the slices homogenized in 3 ml. 0·25 M sucrose; 3 mM $CaCl_2$.

For the estimation of incorporation into total protein, 0·5 ml. of the homogenate was mixed with 0·5 ml. of 1 N $HClO_4$, centrifuged, and the precipitate washed four times with 2 ml. 0·5 N $HClO_4$. The final pellet was dissolved in 1 N NaOH and a fraction taken for protein estimation. Another fraction was counted in a scintillation counter (Packard, Model 3375).

Nuclei were isolated from 2 ml. of the homogenate and fractionated as above omitting the $HClO_4$ extraction and DNA estimations. Fractions of the tris-soluble proteins, acid soluble and acidic proteins were counted and their protein content estimated.

The incorporation of each amino acid was calculated as $\mu\mu$ mole incorporated/mg. protein/hr. The calculations were done with an Olivetti Programma 101

computer, using a programme incorporating quench correction data and data correcting for $^{14}C$ counts in the $^{3}H$ channel. The data were obtained using Packard quenched $^{14}C$ and $^{3}H$ standards.

### RESULTS

### *Growth rate of tumours, and nuclear protein content*

Dependent and independent tumours grew at similar rates during the last week of pregnancy, but independent tumours from non-pregnant mice grew more slowly (Table I). During the second week of pregnancy the tumours regularly showed a pause in growth. Only independent tumours were studied at this time all the dependent tumours being obtained during the last week of pregnancy.

TABLE I.—*Protein Content of BR6 Tumours and Tumour Nuclei*
(mg./mg. DNA ± S.E.M.: Number of estimations in parentheses)

| | Time to double volume (days) | Total protein | Histones | Acidic nuclear proteins | Neutral nuclear proteins |
|---|---|---|---|---|---|
| Dependent tumours (late pregnant mice) | 3–5 (8) | 20·0±1·30 (13) | 1·97±0·18 (10) | 1·51±0·10 (10) | 1·95±0·35 (8) |
| Independent tumours (late pregnant mice) | 3–7 (7) | 29·4±3·2 (9) | 1·08±0·17 (5) | 1·21±0·07 (7) | 0·77±0·11 (7) |
| Independent tumours (mid pregnant mice) | > 20 days (6) | — | 1·56±0·18 (6) | 0·88±0·04 (6) | 1·22±0·18 (6) |
| Independent tumours (non pregnant mice) | 6–20 days (9) | 26·9±2·9 (12) | 1·23±0·10 (9) | 0·89±0·04 (8) | 1·25±0·08 (9) |

The similar growth rates of both types of tumour during the last week of pregnancy permit a direct comparison of dependent and independent tumours under similar conditions of hormonal environment and growth rate. At this time pregnancy dependent tumours had significantly greater amounts of histones ($P < 0{\cdot}01$) acidic nuclear proteins ($P < 0{\cdot}05$) neutral nuclear proteins ($P < 0{\cdot}01$) and total protein ($P < 0{\cdot}02$). The difference in acidic nuclear protein was not very great, and only just significant.

Among pregnancy independent tumours the mean histone content increased with decreasing growth rate, but none of the differences were significant. The mean neutral nuclear protein was lower in the rapidly growing tumours from late pregnant mice then either mid-pregnant mice or non-pregnant mice, but only the latter difference was significant ($P < 0{\cdot}01$). Conversely, the acidic nuclear protein content was significantly higher in the rapidly growing tumours than either group of slowly growing tumours ($P < 0{\cdot}01$). It seems, therefore, that in these tumours the nuclear protein content was related to growth rate. The dependent tumours did not fit into this pattern, having the highest amounts of histone and neutral nuclear protein.

### *Incorporation of amino acids*

The rate of incorporation of both tryptophan and lysine into the protein fractions was approximately linear up to 90 min. (Fig. 1). Both puromycin and zero incubation time controls had less than 10% of the radioactivity found in

FIG. 1.—Incorporation of $^{3}H$-tryptophan (●——●) and $^{14}C$ lysine (○——○) into total proteins, histones and acidic nuclear proteins of BR6 mammary tumours. Tumour slices were incubated for the times indicated and specific incorporation determined as described in the text.

incubated preparations. The specific incorporation ($\mu\mu$ moles/mg./hr) was calculated after subtraction of the c.p.m. in the zero incubation time controls.

The total protein, acidic, and neutral nuclear proteins all incorporated more tryptophan than lysine, while the acid soluble proteins incorporated more lysine than tryptophan (Table II). This was consistent with the relatively high lysine content of histones, which are extracted with acid soluble fraction. However, the ratio of tryptophan : lysine incorporation in the acid soluble protein was very variable. This may have been due to contamination of the histone fraction with neutral nuclear protein, which had very high tryptophan/lysine incorporatio ratios. On the assumption that histones contain negligible amounts of tryptophan, the incorporation data indicate that about 10% of the acid soluble fraction consisted of non-histone protein. This degree of contamination is unlikely to affect the comparisons made in the previous section concerning histone content.

Independent tumours from pregnant mice incorporated more tryptophan ($P < 0{\cdot}02$). but not lysine into neutral nuclear proteins (Table II). No other

TABLE II.—*Incorporation of $^{3}H$ Tryptophan and $^{14}C$ Lysine into Proteins of BR6 Tumours*

($\mu\mu$ moles incorporated/hr/mg. protein $\pm$ S.E.M. and $\mu\mu$ moles incorporated/hr/mg. D.N.A.*) (Number of estimations given in parentheses)

| | | Total proteins | | Histones | | Acidic nuclear proteins | | Neutral nuclear proteins | |
|---|---|---|---|---|---|---|---|---|---|
| | | Tryptophan | Lysine | Tryptophan | Lysine | Tryptophan | Lysine | Tryptophan | Lysine |
| Dependent tumours (6) | Incorporation/mg. protein | 18·9±1·23 | 11·20±1·13 | 0·76±0·17 | 2·40±0·33 | 3·10±0·42 | 1·72±0·17 | 7·10±1·99 | 1·97±0·66 |
| | Incorporation/mg. DNA* | 378·0 | 224·0 | 1·49 | 4·73 | 4·18 | 2·60 | 13·80 | 3·85 |
| Independent tumours from late pregnant mice (4) | Incorporation/mg. protein | 27·45±3·36 | 12·30±1·34 | 2·44±0·58 | 3·92±0·98 | 6·98±0·62 | 2·95±0·47 | 19·27±2·70 | 4·10±2·61 |
| | Incorporation/mg. DNA* | 808·5 | 361·6 | 2·64 | 4·24 | 7·84 | 3·30 | 14·80 | 3·15 |
| Independent tumours from non-pregnant mice (6) | Incorporation/mg. protein | 24·77±4·12 | 14·63±1·70 | 2·35±0·29 | 4·59±0·99 | 5·64±1·05 | 3·23±0·36 | 8·34±2·40 | 3·82±0·66 |
| | Incorporation/mg. DNA | 666·3 | 393·5 | 2·90 | 5·64 | 5·02 | 2·87 | 10·44 | 4·80 |

(* The mean incorporation/mg. protein was multiplied by the mean content of protein/mg. DNA from Table I)

significant differences in rates of incorporation were found between independent tumours from pregnant and non-pregnant mice.

Dependent tumours tended to incorporate less amino acid-mg. protein in each protein fraction than independent tumours. In tumours from pregnant animals these differences were significant for incorporation of tryptophan, but not lysine, into total protein ($P < 0 \cdot 05$) acid soluble nuclear proteins ($P < 0 \cdot 01$) and neutral nuclear proteins ($P < 0 \cdot 01$). Incorporation of both tryptophan and lysine into acidic nuclear proteins was significantly higher in independent tumours from pregnant mice ($P < 0 \cdot 01$, $P < 0 \cdot 05$ respectively). Comparing dependent tumours with independent tumours from non-pregnant mice, no significant difference was found in incorporation into total protein or neutral nuclear protein. Incorporation of trytophan into acid soluble nuclear proteins ($P < 0 \cdot 01$) and lysine into acidic nuclear proteins ($P < 0 \cdot 05$) were significantly higher in the independent tumours.

Since independent tumours contain more total protein/mg. DNA than dependent tumours (Table I) the difference in rate of incorporation/cell would be greater than specific activities indicate. Table II shows the rate of incorporation/mg. DNA, obtained by multiplying mean specific activities by the mean content/mg. DNA of the various proteins (Table I). Expressed this way, the data suggest the incorporation per cell of amino acids into histones and neutral nuclear proteins does not differ very much among the tumours, although incorporation into acidic proteins seems to be highest in independent tumours from pregnant mice.

### DISCUSSION

It has been suggested that histones are involved in the masking of genes (Allfrey, Littau and Mirsky, 1964) and that acidic nuclear proteins act as gene activators (Paul and Gilmour, 1968) possibly by interaction with histones. Much of the evidence in favour of these views comes from the effects of histones and acidic proteins on the template properties of DNA in cell free systems (Wang, 1968). Indirect evidence may also be adduced; for example, Stedman and Stedman (1944) and Mirsky and Pollister (1946) reported that the non-histone protein content of nuclei from rapidly growing tissues and embryos was higher than in slowly growing adult tissue. Rapidly growing rat mammary tumours have more acidic protein than static tumours (Smith *et al.*, 1966). Conversely, low levels of histone have been noticed in rapidly growing tissues, including tumours (Umana, Updike, Randall and Dounce, 1964). Despite the impurity of our " histone " fractions, the present result for independent tumours support the view that concentrations of acidic nuclear proteins are correlated, and histones inversely correlated, with growth rate. However, dependent tumours had higher histone content than any independent tumours, despite their rapid rate of growth. Obviously growth is only one expression of gene activity, and the low histone content of independent tumours may indicate that the chromatin is less effectively masked than that of dependent tumours. The histone content of independent tumours was actually less than that of normal mouse tissues, which ranged from 1·7 to 2·4 mg./mg. DNA for liver, kidney, uterus and spleen (personal observations). Dependent tumours had histone values within this range. Such histone deficiency might explain why the independent tumours had a higher rate of protein synthesis, higher levels of protein and of some enzymes and co-factors than dependent tumours (King,

1968; Smith and King, 1966). Indeed, wherever differences have been found between hormone dependent and independent tumours, whether in enzyme activity, glycolysis, respiration or rates of steroid metabolism, it has been the independent tumours which had the higher levels (King, 1968). It is tempting to suppose they would also have lower histone content.

Oestrogen and androgen sensitive tissues, such as uterus and prostate take up and retain the hormone in their nucleus, while insensitive tissues do not (Jensen and Jacobsen, 1962; King, Gordon and Inman, 1965; Mainwaring, 1969). Similarly, oestrogen independent tumours do not retain oestradiol as well as dependent tumours (Mobbs, 1966, Steggles and King, 1968). It has been suggested that these hormones act by interaction with acidic nuclear protein receptors, which, by interaction with histones, cause derepression (King, 1968; King, Gordon and Steggles, 1969). If the nuclear site of action of the hormones were already unmasked through histone deficiency the hormone would no longer be necessary, and the hormone-receptor complex might be unable to bind to the chromatin. In this way histone deficiency, either on a gross scale, as appears to be the case with BR6 tumours, or on a smaller but more specific scale, could account for hormone independence and the lack of hormone binding in independent tumours. As a corollary to this, tumours deficient in histones might be expected to respond poorly to hormone treatment. The low levels of histone in tumours reported by Umana *et al.* (1964) may reflect a general property of autonomous tumours; the poor response of most tumours to hormones is well known (Potter, 1964).

The differences observed in the amounts of neutral protein in tumours are difficult to interpret because the nature of these proteins is unknown. The specific activities of these proteins were quite different from those of total tumour protein so it seems unlikely that they represent simply cytoplasmic contamination. One possibly is that this fraction contains nuclear ribosomal protein (Frenster *et al.*, 1960).

The rate of incorporation of amino acids into the nuclear proteins was not clearly related to the amounts of protein present, nor to the growth rate of the tumours. The differences in the content of nuclear protein in the different tissues may therefore have been due to differences in relative rates of degradation rather than synthesis of the proteins. However, the mechanism and possible role of nuclear protein turnover in nuclear function is as yet obscure (Busch, Steele, Hnilica and Taylor, 1964).

### REFERENCES

Allfrey, V. G., Littau, V. C. and Mirsky, A. E.—(1964) *J. Cell Biol.*, **21**, 213.

Bonner, J. and Ts'o, P. (editors)—(1964) 'The Nucleohistones'. San Francisco, London, Amsterdam (Holden Day, Inc.).

Burton, K. A.—(1956) *Biochem. J.*, **62**, 315.

Busch, H., Steele, J., Hnilica, L. S. and Taylor, C.—(1964) in 'The Nucleohistones', edited by J. Bonner and P. Ts'o. San Francisco, London, Amsterdam (Holden Day, Inc.) pp. 242–245.

Frenster, J. H., Allfrey, V. G. and Mirsky, A. E.—(1960) *Proc. natn. Acad. Sci. U.S.A.*, **46**, 432.

Foulds, L.—(1949) *Br. J. Cancer*, **3**, 345.

Jensen, E. V. and Jacobsen, H. I.—(1962) *Recent Prog. Horm. Res.*, **18**, 387.

King, R. J. B.—(1968) in ' Prognostic Factors in Breast Cancer ', edited by A. P. M. Forrest and P. B. Kunkler. Edinburgh and London (E. and S. Livingstone Ltd.) pp. 354–363.

King, R. J. B., Gordon, J. and Inman, D. R.—(1965) *J. Endocr.*, **32**, 9.

King, R. J. B., Gordon, J. and Steggles, A. W.—(1969) *Biochem. J.*, **114**, 649.

Lowry, O. H., Rosebrough, N. J., Farr, A. L. and Randall, R. J.—(1951) *J. biol. Chem.*, **193**, 265.

Mainwaring, W. I. P.—(1969) *J. Endocr.*, **44**, 323.

Mirsky, A. E. and Pollister, A. W.—(1946) *J. gen. Physiol.*, **30**, 117.

Mobbs, B. G.—(1966) *J. Endocr.*, **36**, 409.

Paul, J. and Gilmour, R. S.—(1968) *J. molec. Biol.*, **34**, 305.

Potter, V. R.—(1964) *Cancer Res.*, **24**, 1085.

Smith, J. A. and King, R. J. B.—(1966) *J. Endocr.*, **35**, 281.

Smith, J. A., King, R. J. B., Meggitt, B. F. and Allen, L. N.—(1966) *Br. J. Cancer*, **20**, 335.

Stedman, E. and Stedman, E. (1944) *Nature, Lond.*, **153**, 500.

Steggles, A. W. and King, R. J. B.—(1968) *Eur. J. Cancer*, **4**, 395.

Umana, R., Updike, S., Randall, J. and Dounce, A. V.—(1964) in ' The Nucleohistones ', edited by J. Bonner and P. Ts'o. San Francisco, London, Amsterdam (Holden Day Inc.) pp. 200–229.

Wang, T. Y.—(1968) *Expl Cell Res.*, **53**, 288.